# Analysis of the Influence of Activated Carbons’ Production Conditions on the Porous Structure Formation on the Basis of Carbon Dioxide Adsorption Isotherms

**DOI:** 10.3390/ma15227939

**Published:** 2022-11-10

**Authors:** Mirosław Kwiatkowski, Xin Hu, Piotr Pastuszyński

**Affiliations:** 1 Faculty of Energy and Fuels, AGH University of Science and Technology, 30 Mickiewicza Avenue, 30-059 Krakow, Poland; 2 College of Chemistry and Life Sciences, Zhejiang Normal University, Jinhua 321004, China

**Keywords:** adsorption, micropores, chemical activation, carbonaceous adsorbents

## Abstract

The results of a study of the impact of activation temperature and the mass ratio of the activator to the carbonised precursor on the porous structure of nitrogen-doped activated carbons obtained from lotus leaves by carbonisation and chemical activation with sodium amide (NaNH_2_) are presented. The analyses were carried out via the new numerical clustering-based adsorption analysis, the Brunauer–Emmett–Teller, the Dubinin–Raduskevich, and the density functional theory methods applied to carbon dioxide adsorption isotherms. Carbon dioxide adsorption isotherms’ analysis provided much more detailed and reliable information about the pore structure analysed. The analyses showed that the surface area of the analysed activated carbons is strongly heterogeneous, but the analysed activated carbons are characterised by a bimodal pore structure, i.e., peaks are clearly visible, first in the range of pore size from about 0.6 to 2.0 nm and second in the range from about 2.0 to 4.0 nm. This pore structure provides optimal adsorption performance of carbon dioxide molecules in the pore structure both for adsorption at atmospheric pressure, which requires the presence of narrow pores for the highest packing density, as well as for adsorption at higher pressures, which requires the presence of large micropores and small mesopores. However, there are no micropores smaller than 0.5 nm in the analysed activated carbons, which precludes their use for carbon dioxide adsorption for processes conducted at pressures less than 0.01 MPa.

## 1. Introduction

The greenhouse effect is a natural process that enables life on Earth by absorbing heat provided by solar radiation, and then releasing it in the form of emission of long-wave radiation to the components of the atmosphere and secondary emission towards the Earth’s surface [1,2]. It is a natural and desired process through which air near the Earth’s surface gains the appropriate temperature [3]. The heat required to live on Earth is held in the atmosphere by a layer of greenhouse gases, such as carbon dioxide, methane, freons, ozone, hydrocarbons, and oxides of nitrogen. However, due to human activity, more radiation builds up in the atmosphere, causing a global problem known as global warming. Climatic models predict that global temperatures will increase by about 6 °C by the year 2100. The spread of global warming is linked to increased greenhouse gas emissions in the atmosphere because of rapid urbanisation and increased energy and industrial consumption [3]. The negative impact of global warming is manifested through melting glaciers, rising ocean temperatures, and increased frequency of extreme weather events, including heat waves [3].

Currently, the best way to reduce the amount of carbon dioxide (CO_2_) in the atmosphere is through carbon capture and storage. At present, four main methods for sequestering CO_2_ are being developed: pre-combustion, post-combustion, oxyfuel, and chemical looping combustion [3]. However, in the case of heavy industrial plants, such as steel mills, cement plants, or power plants, which derive energy from the combustion of fossil fuels, the most used method is the capture of carbon dioxide from the flue gases after the combustion process [4,5].

In large-scale practical applications, the CO_2_ sequestration method should be characterised by low energy input necessary for the process, low production cost, easy availability of materials, and their high service life, as well as high efficiency of the CO_2_ absorption process and no negative impact on the natural environment. One of the most common ways of capturing carbon dioxide is chemical absorption through aqueous mixtures of organic amines [6]. The downside of this method is the use of highly volatile and corrosive components of the absorbing mixture and the need to supply significant amounts of energy in the amine regeneration process. However, the most promising method for sequestering carbon dioxide, which can be widely applied in practice, is the adsorption method [7,8,9,10,11]. Carbon dioxide sequestration by adsorption is the process of separating CO_2_ from the combustion gas by adsorbing it to the surface of the adsorbent. The adsorption process is performed to saturate the adsorbent with CO_2_. After that, at the desorption stage, the adsorbed gas is released under the influence of a specified temperature. The adsorbents used in the CO_2_ sequestration method are zeolites, metal oxides, silica gels, ion-exchange resins, and especially activated carbons [12].

The most relevant adsorbents used in carbon sequestration technologies have proven to be activated carbons derived from biomass [13,14,15,16,17,18,19,20,21,22,23,24]. This is due to the widespread availability of raw materials for their production and their renewability, significant strength, high adsorption capacity, stability, and ease of regeneration [13,14,15,16,17,18,19,20,21,22,23,24].

The development of advanced adsorption technologies requires activated carbons with very high adsorption capacity as well as suitable mechanical properties. Therefore, new solutions are being sought in the field of activated carbon production technologies by chemical activation [25,26,27]. However, it should be noted that the optimal method of chemical activation should not only develop a porous structure, but also, through appropriate reagents, modify the properties of the adsorbents. The method of doping activated carbons with nitrogen shows great potential, because even with a small amount of nitrogen, activated carbons significantly increases its carbon dioxide adsorption capacity [28]. Nitrogen present in the activated carbon structure changes the surface electrostatic potential of adjacent carbon atoms, increasing its local electron density, which has the effect of enhancing the interaction between the surface of activated carbon and adsorbed CO_2_ [28,29,30]. The first attempts to apply nitrogen atoms to the microporous structure of activated carbon were carried out with the use of gaseous ammonia and a pre-prepared activated carbon precursor. The disadvantage of this method was the necessity to conduct the process in two stages and its energy consumption, ammonia toxicity, and low efficiency. As a source of nitrogen and an activator in the production of activated carbons, sodium amide (NaNH_2_) is used. This compound allows the activation process to be conducted at a lower temperature than the activators used so far and has a lower toxicity than ammonia [31,32].

Despite the widespread use of nitrogen in the analysis of adsorption processes, it is increasingly emphasised that the use of this adsorbate, especially for the analysis of microporous materials, leads to erroneous estimates of structure parameters due to the hysteresis effects observed during nitrogen (N_2_) adsorption [33,34]. This is due, among other things, to the fact that the value of the so-called sitting area of the nitrogen molecule of 0.162 nm^2^ assumes that the nitrogen molecule adsorbs flat on the adsorbent surface. In fact, functional groups interacting with the quadrupole moment of nitrogen have been shown to lead to a change in the orientation of the adsorbed nitrogen molecule. Consequently, the sitting cross-sectional area of a nitrogen molecule can be much smaller than the commonly assumed value, which consequently can lead to a significant error in the estimation of the specific surface area [33,34].

Nitrogen molecules also cannot enter the narrowest micropores, i.e., less than 0.45 nm-wide due to kinetic constraints. Carbon dioxide molecules at room temperature do not have this type of limitation due to their smaller kinetic diameter, compared to nitrogen molecules. Consequently, a commonly used complement to porous structure analyses based on isotherms of nitrogen adsorption at temperature is the study based on isotherms of adsorption of carbon dioxide (CO_2_) at 273 K [35,36,37,38].

Adsorption of CO_2_ at atmospheric pressure requires the presence of narrow pores, i.e., less than 1 nm, which are essential for optimal CO_2_ adsorption performance at atmospheric pressure [39]. Therefore, the evaluation of the ability of microporous carbon materials in capturing CO_2_ at atmospheric pressure, i.e., the total adsorption capacity, is determined by the volume of narrow micropores [39,40,41].

## 2. Materials and Methods

The results of a study which yielded nitrogen-doped activated carbons prepared from lotus leaves via carbonisation and chemical activation with NaNH_2_ were presented by Kwiatkowski and Hu [42], carried out on the basis of on nitrogen adsorption isotherms. Since the activated carbons obtained from lotus leaves analysed by Kwiatkowski and Hu were characterised by a microporous structure [42], the concept of a significant extension of the conducted studies by a comprehensive analysis was based on carbon dioxide adsorption isotherms obtained at 273 K [43]. 

The current study can provide more insights on the effect of activation parameters on porous structure formation for carbonaceous adsorbents and hence can help to design more effective materials for removing CO_2_ from combustion flue gas. This can reduce significant amounts of CO_2_ and be beneficial to Earth’s environment and sustainable development.

The new series of analyses were carried out via the new numerical clustering-based adsorption analysis (LBET) [44,45,46,47,48,49], and the Brunauer–Emmett–Teller (BET) [50], the Dubinin–Raduskevich (DR) [51], and the density functional theory (DFT) [52,53] methods. The porosity of activated carbons was measured with a built-in DFT method in Quantachrome autosorb-1, that assumed slit pores with flat and energetically uniform graphitic walls.

## 3. Discussion of the Obtained Results

**Figure 1:**
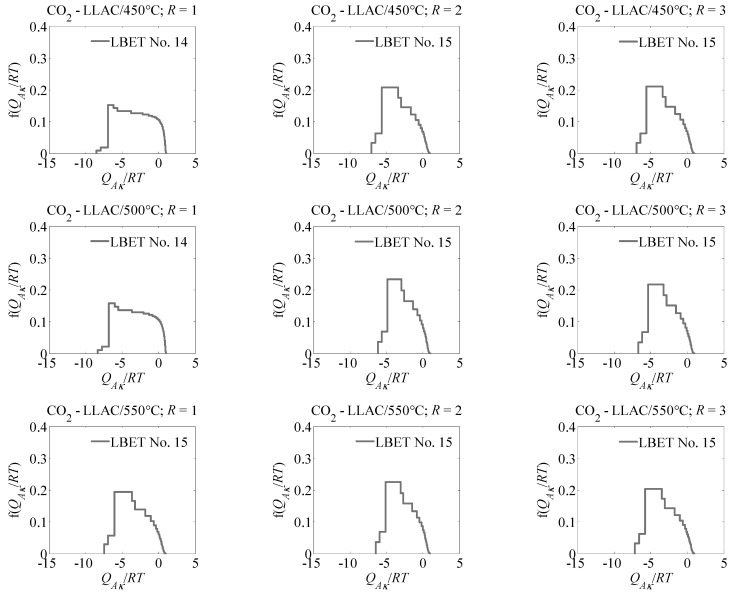
Adsorption energy distributions on the first layer, obtained by the LBET method based on the nitrogen adsorption isotherms.

**Figure 2:**
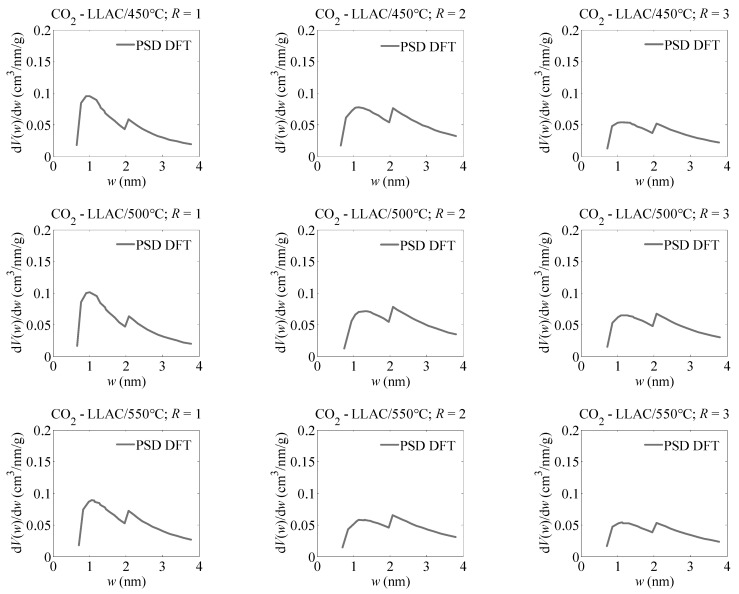
The pore size distributions obtained for the analysed active carbons using the DFT method.

The results of the analyses performed are presented collectively in the Table 1, as well as in the Figure 1 and Figure 2. On the basis of the aforementioned results, it can be concluded that the analysed samples of activated carbons obtained from lotus leaves are characterised by relatively low values of the specific surface area determined by means of the BET equation. As can be seen, in the case of the sample obtained at 450 °C, with an increase in the mass ratio of the activator to the char, *R* from 1 to 2, the value of the specific surface area increased. With a further increase in the mass ratio *R* = 3, however, a significant decrease in the value of this parameter occurred. On the other hand, for the sample obtained at 500 °C, a slight difference can be observed between the *S_BET_* surface values determined for samples obtained at *R* equal to 1 and 2. Meanwhile, in the case of mass ratio *R* = 3, a significant decrease in the specific surface value *S_BET_* can be observed analogously as before.

In the case of the sample obtained at 550 °C, a significant decrease in the value of the specific surface area was observed already at mass ratio, *R*, equal to 2, in comparison with the value obtained at *R* = 1, and successively, a further decrease in the value of this parameter at mass ratio *R* = 3. As can be seen, the values of specific surface area calculated via the BET equation are correlated with the values of total pore volume, *V_total DFT_*, determined via the DFT method. The micropore volume values *V_micro DR_* and *V_total DFT_* achieved via the DR and DFT methods also indicate that as the mass ratio value, *R*, increases, the micropore volume decreases.

Significant information about the analysed structure was obtained using the LBET method; namely, for the analysed activated carbon samples, the average values of the first-layer adsorbed volume parameter, *V_hA_*, were obtained, and for the sample prepared at 450 °C, the values of the parameter *V_hA_* decreased with increasing mass ratio, *R*. On the other hand, for the sample obtained at *T* = 500 °C, the value of the *V_hA_* parameter increased at a mass ratio of *R* = 2 and then decreased at a mass ratio of *R* = 3. For the sample prepared at 550 °C, the same situation occurred as for the sample obtained at 500 °C.

The values of energy parameters *Q_Ak_*/*RT* and *B_C_* calculated for the analysed samples indicate preferential conditions for the formation of extended clusters of adsorbate molecules, and the values of heterogeneity parameters, *h*, indicate that the surface of the studied samples is geometrically and energetically heterogeneous. In turn, the values of geometric parameters *α* and *β* indicate that high and forking clusters of carbon dioxide molecules are adsorbed in the pores of the analysed samples.

The values of the dispersion of the fitting error, *σ_e_*, point to very good fitting of the theoretical isotherms for empirical data, but as can be seen in Table 1, relatively low identification rates were obtained for all the analysed activated carbons, indicating the existence of some significant deviation of the pore structure from the assumptions of the theory underlying the LBET method. The shapes of the adsorption energy distributions on the first layer determined via the LBET method indicate the existence of a wide energy range of primary adsorption sites. In addition, as can be seen with the growth in the value of the mass ratio, *R*, to a value equal to 2, the width of the adsorption energy distributions on the surface of the tested adsorbents noticeably decreased.

Similar results were achieved via the DFT study, especially in obtaining the shapes of pore size distributions (Figure 2). Based on pore size distributions determined via the DFT method, it has been presented that the analysed activated carbons were characterised by a bimodal pore structure, i.e., peaks were clearly visible, first in the range of pore size from about 0.6 to 2.0 nm and second in the range from about 2.0 to 4.0 nm. 

This pore structure provides optimal adsorption performance of carbon dioxide molecules in the pore structure both for adsorption at atmospheric pressure, which requires the presence of narrow pores, preferably below 0.7–0.8 nm for the highest packing density, as well as for adsorption at higher pressures, which requires the presence of large micropores (1 to 2 nm) and small mesopores (pores larger than 2 nm). It is worth noting the absence of the smallest pores, i.e., smaller than 0.5 nm, which excludes the application of the obtained activated carbons for carbon dioxide adsorption for processes carried out at a pressure of 0.01 MPa. 

The N-doped porous carbonaceous adsorbent activated by sodium amide analysed in this publication has comparable CO_2_ adsorption performance to many typical adsorbents, such as activated carbons [54,55], metal-organic frameworks [56], covalent organic frameworks [57], porous aromatic frameworks [58], and porous polymers [59].

## 4. Conclusions

The use of waste biomass to produce carbonaceous adsorbents for application in carbon dioxide capture processes is in keeping with the concept of a circular economy. Activated carbons derived from biomass are not only environmentally friendly materials, thanks to the reduction of waste generation, but they are also relatively inexpensive due to the possibility of easy acquisition of raw material. As demonstrated in the present work, the morphological structure and porous structure of activated carbons prepared from lotus leaves via pyrolysis and chemical activation provide, thanks to the specific bimodal porous structure, good conditions for the carbon dioxide adsorption process both at atmospheric pressure and high pressures. Also noteworthy is the large availability of raw material and the relatively low cost of both the raw material and the chemical activator, which creates prospects for the development of the production of activated carbons by this method on an industrial scale. As the analyses showed, the LBET method allowed obtaining a wider range of information on the porous structure of the analysed activated carbons compared to the BET and DR methods. However, the complete range of information about the analysed pore structure is only provided by the simultaneous use of LBET and DFT methods, which are complementary to each other. 

## Figures and Tables

**Table 1:** The results of the analysis of a porous structure of activated carbons, based on carbon dioxide adsorption isotherms, using the BET, DR, and LBET methods.

CO_2_	450 °C			500 °C			550 °C		
*R*	1	2	3	1	2	3	1	2	3
*S_BET_*	287.76	309.97	213.21	306.21	305.12	266.75	311.12	251.41	217.94
*V_micro DR_*	0.1214	0.1096	0.0716	0.1282	0.1003	0.0850	0.1121	0.0738	0.0715
*V_micro DFT_*	0.1069	0.1012	0.0683	0.125	0.0877	0.0829	0.1055	0.0757	0.0713
*V_total DFT_*	0.1593	0.1794	0.1220	0.1686	0.1706	0.1537	0.1745	0.1473	0.1277
LBET No.	14	15	15	14	15	15	15	15	15
*V_hA_* (cm^3^/g)	0.473	0.112	0.107	0.512	0.746	0.561	0.542	0.582	0.417
*Q_Ak_/RT*	–8.54	–7.06	–6.95	–8.35	–6.18	–6.72	–7.49	–6.44	–7.16
*B_C_*	1.00	1.00	1.00	1.00	1.00	1.00	1.00	1.00	1.00
*Z_A_*	0.718	0.633	0.628	0.707	0.584	0.614	0.658	0.598	0.639
*h*	9	9	9	9	9	9	9	9	9
*α*	0.98	0.88	0.88	0.98	0.89	0.88	0.89	0.88	0.87
*β*	1.710	1.83	1.85	1.79	1.80	1.81	1.78	1.91	1.88
*σ_e_*	0.077	0.13	0.078	0.069	0.076	0.099	0.09	0.13	0.11
*w_id_*	0.02	0.03	0.03	0.03	0.04	0.03	0.03	0.03	0.02

## Data Availability

The data presented in this work can be made available upon request.
